# Effect of Various Protocols of Pre-Emptive Pulpal Laser Analgesia on Enamel Surface Morphology Using Scanning Electron Microscopy: An Ex Vivo Study

**DOI:** 10.3390/biomedicines11041077

**Published:** 2023-04-03

**Authors:** Ani Belcheva, Elitsa Veneva, Reem Hanna

**Affiliations:** 1 Department of Pediatric Dentistry, Faculty of Dental Medicine, Medical University-Plovdiv, 3 Hristo Botev Blvd, 4000 Plovdiv, Bulgaria; 2 Department of Surgical Sciences and Integrated Diagnostics, Laser Therapy Centre, University of Genoa, Viale Benedetto XV, 6, 16132 Genoa, Italy; 3 Department of Oral Surgery, King’s College Hospital NHS Foundation Trust, Denmark Hill, London SE5 9RS, UK; 4 Department of Restorative Dental Sciences, UCL-Eastman Dental Institute, Faculty of Medical Sciences, Rockefeller Building, London WC1E 6DE, UK

**Keywords:** light-induced therapy, scanning electron microscopy, light-responsive molecule, anaesthesia, photodiagnostics, enamel ablation, photobiology, Er:YAG, enamel–light interaction, pulp anaesthesia, restorative dentistry

## Abstract

Achieving local anaesthesia for various clinical dental applications is a challenge that we encounter in our daily practice. Pre-emptive pulpal laser analgesia (PPLA) treatment strategy could be a promising non-pharmacological modality. Hence, our ex vivo laboratory study is aimed at evaluating the changes in enamel surface morphology when irradiated with various published PPLA protocols using scanning electron microscopy (SEM). To do so, 24 extracted healthy human permanent premolar teeth were collected, and each tooth was divided into equal halves randomised into six groups. The following laser parameter protocols based on published protocols of clinical Er:YAG laser-induced PPLA were randomly assigned for each group: 0.2 W/10 Hz/3 J/cm^2^ (Group A—100% water spray; Group B—no water); 0.6 W/15 Hz/10 J/cm^2^ (Group C—100% water spray; Group D—no water); 0.75 W/15 Hz/12 J/cm^2^ (Group E—100% water spray; Group F—no water); 1 W/20 Hz/17 J/cm^2^ (Group G—100% water spray; Group H—no water). Each sample was irradiated at an angle of 90° to the dental pulp, with a sweeping speed of 2 mm/s for a 30 s exposure time. Our results have shown, for the first time, no alteration to the mineralised tooth structure when irradiated with the following protocols: 0.2 W/10 Hz/3 J/cm^2^ with 100% water spray or without water spray with an irradiated area fixed at a 10 mm tip-to-tissue distance, sweeping motion with 2 mm/s speed of movement; average power output of 0.6 W/15 Hz/10 J/cm^2^, maximum water cooling of 100%, tip-to-tooth distance fixed at 10 mm, 30 s exposure time, sweeping motion with 2 mm/s speed of movement. The authors concluded that the current available proposed PPLA protocols in the literature might cause an alteration to the enamel surface. Hence, future clinical studies are warranted to validate our study’s PPLA protocols.

## 1. Introduction

Achieving local anaesthesia for various clinical applications is a challenge that we encounter in our daily practice. In this context, pre-emptive pulpal laser analgesia (PPLA) treatment strategy could be a promising non-pharmacological modality, considering its non-thermogenic biomodulation of the dental pulp responsiveness by reducing the pulpal nociceptive nerve impulse formation; it is obtainable in a low-energy-level irradiation form [1,2]. Hence, PPLA can be utilised in various clinical applications, especially in restorative dentistry, where Er:YAG (2940-nm) is one of the most commonly utilised wavelengths. Fluence plays a crucial role in achieving an optimal outcome of Er:YAG laser-induced PPLA [3,4], as well the photo-acoustic effect of pulsed lasers [3].

Nevertheless, currently, there is no established consensus for a standardised PPLA protocol due to either a lack of reliable laser parameters or unreported essential laser parameters. Many concepts have been hypothesised, with one related to the utilisation of low fluences of laser pulses altering the cell membrane behaviour of the pulpal nerve fibres by coinciding with their natural resonance frequency (15–20 Hz) [5]. This leads to hyperpolarisation and loss of the nociceptive impulse conduction.

Walsh [1] suggested that in order to achieve an effective analgesia, the use of sub-ablative pulse energies at an appropriate frequency is essential, and the following protocols for the erbium lasers and Nd:YAG, respectively, were proposed: 60–120 mJ/pulse (defocused, with water spray), at a frequency between 10 and 30 Hz (preferably 20 Hz), applied for at least 30 s; 50–100 mJ/pulse (defocused, without water spray), at a frequency of 20 Hz, once again applied for 30 s at each of the line angles of the tooth.

Many studies have agreed that in order to achieve a good PPLA, it is necessary to take advantage of low energy and fluences with a circular irradiation motion of the tooth’s neck area at a distance of 3–10 mm [6,7,8,9,10]. Nevertheless, the current literature shows conflicting findings. A study by Bjordal et al. [11] utilised 7.5 J/cm^2^, whereas another study, conducted by Angelieri et al. [12], proposed greater levels of fluence within the range 5–20 J/cm^2^ for severe pain. A fluence of 35 J/cm^2^ was suggested by another group of investigators to reduce orthodontic pain, since a level of only 5 J/cm^2^ was not found to be effective; however, in the currently available literature, conflicting results are reported for similar protocols [13].

Moreover, the morphology and nature of the tooth structure in terms of enamel and dentine need to be taken into consideration during the formulation of PPLA protocol. An evaluation of laser irradiation’s effect on surface enamel must be based on the knowledge of its natural morphology. In humans, the outermost layer of the enamel surface is composed of columns of hydroxyapatite crystals arranged parallel to each other and perpendicular to the enamel periphery, designated prismless enamel [13].

The prismless enamel was initially identified as an area that has no prism boundaries due to the parallel arrangement of the crystals and has a thickness of about 5–25 μm [13]. However, this definition is not functional due to the frequent sporadic presence of unclear and/or incorrect prism structures in areas otherwise considered aprismatic [14,15,16,17]. Given the variety in frequency, structure and thickness of the prismless enamel in human teeth [18], the development of an index evaluating laser-induced effect on the enamel surface appears to be obsolete. On the basis of this theoretical rationale, we have identified object-based analysis as the most appropriate for the means of our investigation.

PPLA settings are fundamental to induce laser analgesic effects. However, there is a lack of research determining the optimal mid-infrared (IR) energy and parametry to produce more predictable protocol and reliable laser analgesic-based treatment protocol. Hence, we conducted our ex vivo study aiming to examine and evaluate the effects of the currently available protocols for Er:YAG laser-induced PPLA on enamel surface morphology via scanning electron microscopy (SEM). The null hypothesis of our study was to validate that the currently available protocols for Er:YAG laser-induced PPLA initiate morphological changes on the enamel surface. In this context, the study’s objectives are as follows: (1). To establish the most suitable Er:YAG-induced PPLA protocol with effective fluence without enamel morphology changes for future clinical studies; (2). To determine whether low or high fluence has an influence on enamel surface morphology.

## 2. Materials and Methods

### 2.1. Study Design

This was a randomised ex vivo laboratory study, where 24 sound human premolar teeth free of racks, erosion, caries or any structural defect were collected. Then, each tooth was divided into equal halves and the 48 enamel surfaces were randomly assigned into eight groups (Groups A–I) according to the laser protocols illustrated in Table 1. The randomisation process was achieved via a computer-generated permuted block sequence in matched pairs design. All the collected teeth were extracted for orthodontic purposes. Informed written consent was obtained from all the patients and patients’ legal guardians for the purposes of utilising their teeth in research and publications.

### 2.2. Preparation of the Enamel Samples

All the 24 extracted teeth were stored in 0.1% thymol solution prior to commencing the experiments. Then, the teeth were sectioned mesio-distally into two equal halves (48 surfaces), using a diamond-tipped sectorial blade of an “Minosekar-2, Imer” sawing machine. The device is located at the Bulgarian Academy of Sciences, Institute of Physical Chemistry “Rostislaw Kaischew”, Sofia, Bulgaria.

### 2.3. Experimental Design

The enamel surfaces of each tooth that was divided into two halves were irradiated with the same power output, and one half was subjected to 100% water spray cooling, whereas the other half was not. This approach provided a sample from each tooth in two groups of the same power settings while differing in their water-cooling status. This was an equivalent and within-subject control design.

Table 1 summarises the settings of the laser parameters for each group, which were adjusted and modified to adapt properly to each device given its limitations. Pulsed Er:YAG (2940-nm) (LiteTouch™, Light Instruments LTD CAM building, 2nd floor, 4 HaTnufa St. Industrial Zone Yokneam, 2066717, P.O. Box 223, Yokneam 2069203, Israel) was utilised in this study.

The rationale behind choosing different parameter settings recommended for clinical Er:YAG laser-induced PPLA was equivalent to and supported by the literature clinical parameters for Er:YAG laser induction pulpal analgesia of Walsh et al. [3], which were as follows: “subablative pulse energies between 60–120 mJ/pulse (defocused, with water spray), at a frequency ranged between 1–30 Hz (preferably 20 Hz), applied for at least 30 s”.

A study by Walsh et al. [3] highlighted that the optimal effect can be achieved at a pulse frequency of 20 Hz, which appears to align with the resonance frequency needed to temporarily disrupt the action of the Na/K pump, whereas the sweeping motion was at approximately 2–3 mm/second (s), during which 20–30 s exposure time at each line angle can distribute the effect evenly across the dental pulp [3].

### 2.4. Laser Irradiation Process

**Figure 1:**
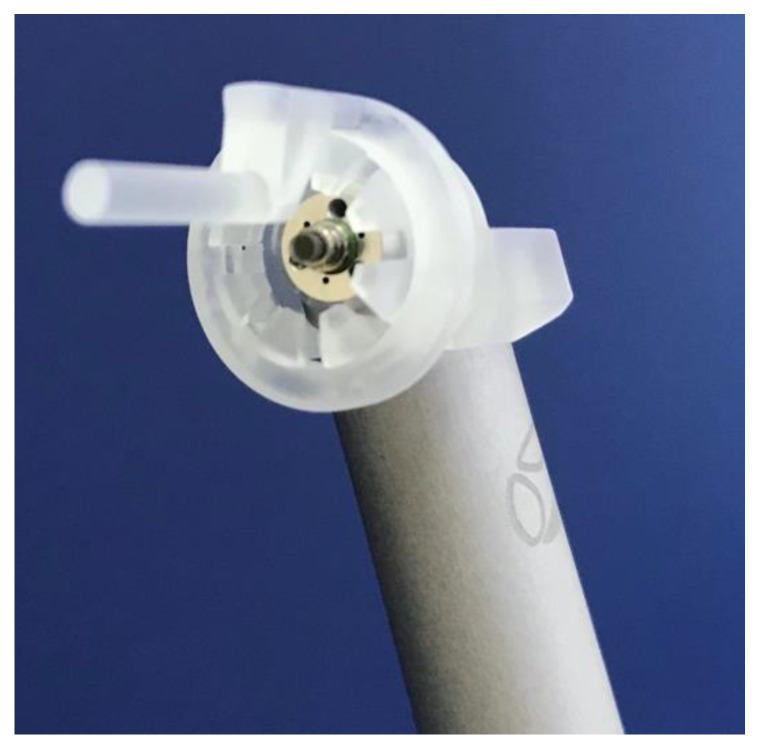
Adjustable rotating spacer, providing tip-to-tissue distance of 10 mm from 1.3 × 6.3 mm sapphire tip, used in this study.

A non-contact hand-piece with a sapphire tip 1.3 × 6.3 mm in diameter was held fixed at a 10 mm distance from the tooth neck by using a spacer designed specifically for the purpose of the present study (Figure 1). The photonic energy of 2940 nm was utilised to irradiate the cervical enamel surface above the cemento-enamel junction (CEJ) perpendicularly towards the dental pulp of each half at a sweeping speed of 2 mm/s for 30 s, in order to evaluate the optimal sub-ablative effect of various literature-recommended PPLA parameters and irradiation areas such as the neck and occlusal surfaces of the teeth [10,19]. Our study PPLA protocol was formulated based on this.

### 2.5. Scanning Electron Microscopy (SEM)

#### 2.5.1. SEM Description

SEM device that was utilised in our study was a JEOL JSM 6390 with an attachment (INCA Oxford) for elemental analysis, allowing the determination of the surface structure, morphology and elemental composition of any materials. The JSM-6390 is a high-performance SEM with a high resolution of 3.0 nm [20]. The customizable GUI interface allows the instrument to be intuitively operated, and the software ensures optimum operation settings. The JSM-6390 specimen chamber can accommodate a specimen of up to 6 inches in diameter. Standard automated features include auto focus/auto stigmator, auto gun (saturation, bias and alignment) and automatic contrast and brightness. The JSM-6390 is used in many varied applications with several options that increase its versatility. SEM has SE (secondary electron), BSE (backscattered electron) and CL (cathodoluminescence) imaging capabilities, and can provide standard-based analyses of major and some minor elements through EDS (energy dispersive spectrometry) [21]. The manufacturer of the SEM-JEOL JSM 6390 is JEOL Ltd. The company’s main office is located in 3-1-2 Musashino, Akishima, Tokyo 196-8558, Japan.

#### 2.5.2. SEM Preparation

After completion of the samples’ irradiation, the prepared samples of each group, as described in Section 2.1, were examined using SEM. Subsequently, the samples were fixed in 2.5% glutaraldehyde solution for 12 h (4 °C), dehydrated (25–100% ethanol in increasing concentrations), dried and then spatter-coated with gold [18]. Finally, all the enamel surfaces were analysed with SEM at ×50, ×100, ×500 and ×1000 magnifications. The SEM preparation, examination and analysis were performed by two independent experienced chemists (SA and IS) at the Department of Physics and Chemistry, Bulgarian Academy of Science, Sofia, Bulgaria. The examined samples were rated on the basis of the absence or presence of surface impact cratering, scaly surfaces or exposed enamel prisms.

## 3. Results

All the SEM analyses were performed after the Er:YAG laser-induced analgesic treatments were completed. Under ×50 magnification, the micrographs of all the samples showed striae of Retzius, where few of them were either masked focally with debris or showed fine cracks and lamella on their surfaces. The debris was found only in the samples that was irradiated without water cooling.

**Figure 2:**
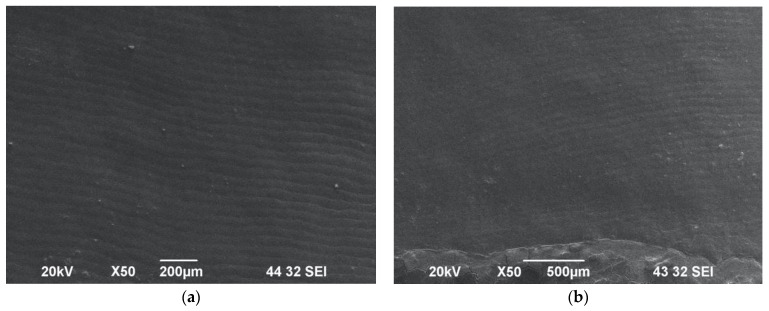
SEM images of enamel surfaces irradiated with 0.2 W/10 Hz/20 mJ/3 J/cm^2^ under ×50 magnification. Group A (**a**) was irradiated without water spray cooling and Group B (**b**) had maximum water spray applied, with both showing an intact enamel surface morphology with striae of Retzius.

**Figure 3:**
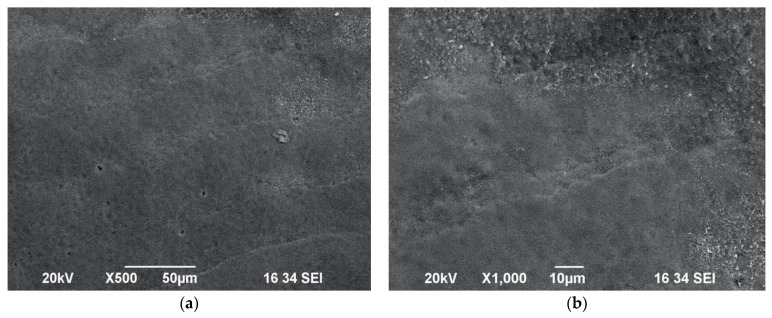
SEM images of tooth surface irradiated with 0.6 W/15 Hz/40 mJ/10 J/cm^2^ with 100% water spray—Group C—under ×500 magnification (**a**) and ×1000 magnification (**b**). Observation of intact enamel surface morphology, showing striae of Retzius.

The laser-irradiated samples in the following groups were examined using SEM, and showed no alteration to the mineralised tooth structure: Group A, Group B (Figure 2a,b), Group C (Figure 3a,b), Group E (Figure 5a,b) and Group G (Figure 7a,b). Nevertheless, samples that were irradiated without water at the lowest used energy of 0.2 W at a frequency of 10 Hz with a fluence of 3 J/cm^2^ were observed to be sub-ablative in Group B, when a 10 mm laser tip-to-tissue distance was employed. Equally, the samples of Groups A and B were irradiated with the protocol 0.2 W/10 Hz/3 J/cm^2^, but those in Group A exposed to 100% water spray showed an absence of surface cratering or melting, whereas the samples in Group B exposed to no water cooling had striae of Retzius clearly visible, which is well illustrated in Figure 2a,b.

**Figure 4:**
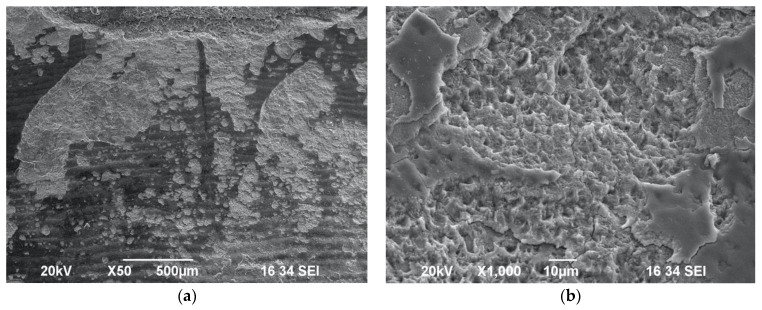
SEM images of enamel surface morphology following irradiation with 0.6 W/15 Hz/40 mJ/ 10 J/cm^2^ without water spray cooling—Group D—under ×50 magnification (**a**) and ×1000 magnification (**b**), showing presence of impact craters and scaly surface.

Interestingly, when samples of Group C were irradiated with the protocol 0.6 W/15 Hz/10 J/cm^2^ with a maximum water spray level of 100%, this resulted in a lack of alteration in the enamel surface morphology—no impact craters or melting surfaces were observed (Figure 3a,b). However, when the same energy settings were applied without water cooling in Group D (Figure 4a,b), the SEM investigation showed the presence of impact craters and scaly surfaces. In this context, it is noteworthy that in order to achieve a sub-ablative laser action at a distance of 10 mm from the target tissue, a maximum water of 100% should be applied with the following protocol: 0.6 W/15 Hz/10 J/cm^2^.

**Figure 5:**
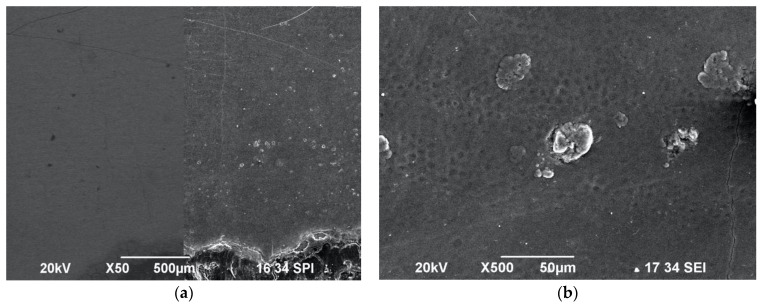
SEM images of enamel surface morphology following irradiation with 0.75 W/15 Hz/50 mJ/12 J/cm^2^ with maximum water spray cooling—Group E—under ×50 magnification (**a**) and ×500 magnification (**b**), showing presence of scattered impact craters.

**Figure 6:**
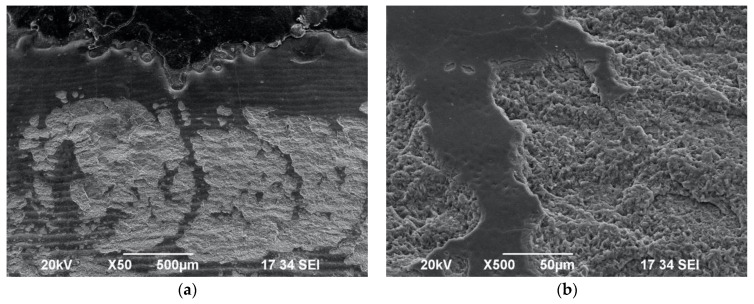
SEM images of enamel surface morphology following irradiation with 0.75 W/15 Hz/50 mJ/12 J/cm^2^ without water spray—Group F—under ×50 magnification (**a**) and ×500 magnification (**b**), showing scaly surface and wide-diameter impact craters.

Laser-induced impact craters and scaly ablated morphology on the enamel surface were observed as well in Group E (Figure 5a,b) and Group F (Figure 6a,b) samples irradiated with the following laser settings: 0.75 W/15 Hz/12 J/cm^2^ with or without maximum water cooling, at a distance of 10 mm from target area. Hence, it can be considered as an ablative protocol.

**Figure 7:**
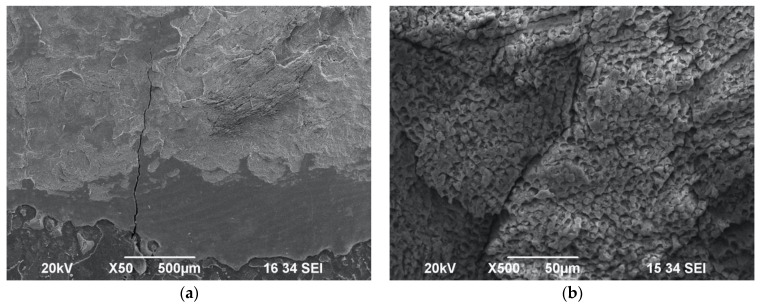
SEM images of enamel surface morphology following irradiation with 1 W/20 Hz/50 mJ/17 J/cm^2^ with maximum water spray cooling—Group G—under ×50 magnification (**a**) and ×500 magnification, (**b**) showing scaly surface and impact craters with exposed prisms.

**Figure 8:**
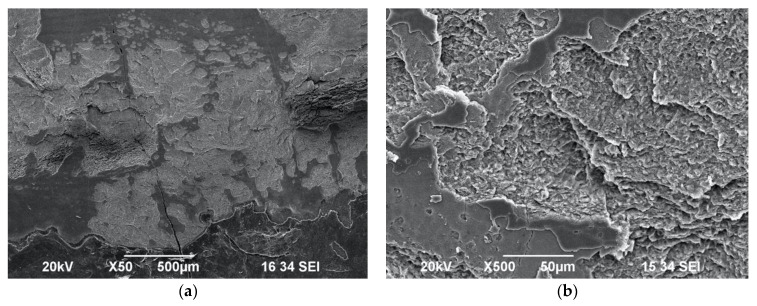
SEM images of enamel surface morphology following irradiation with 1 W/20 Hz/50 mJ/17 J/cm^2^ with no water spray cooling—Group H—under ×100 magnification (**a**) and under ×500 magnification (**b**), showing scaly surface and wide-diameter impact craters with exposed prisms.

The presence of wide scaly surfaces with impact craters and exposed prisms was observed in the samples of Group G (Figure 7a,b) and Group H (Figure 8a,b). Additionally, the impact craters appeared to be of a wider diameter and at a greater depth compared to the Group G samples irradiated with water when the samples in Group H were irradiated with the following laser protocol without water spray: 1 W/20 Hz/17 J/cm^2^.

## 4. Discussion

The data of our ex vivo study demonstrated for the first time that the current published clinical PPLA protocols can cause enamel morphological changes.

The concept of laser-induced analgesia, “photobiomodulation (PBM) of pulpal reactivity”, is developed, contributing to a minimal-to-none use of local anaesthesia, resulting in a reduction in the discomfort experienced by patients during dental restorative intervention, especially cavity preparation.

Many studies have documented that PBM is effective within the optical window of (600–1100 nm) [9,22]. Hence, the mechanism of PBM pulpal reactivity of pulsed mid-IR lasers (Nd:YAG, Er:YAG and Er, Cr:YSGG) is different and employed for a low-level effect in PPLA [10,19,23].

The present study utilised an Er:YAG laser (2940 nm), which coincides with the peak absorption of water. The hydroxyl groups in the hydroxyapatite crystals can absorb some of the generated irradiation, but to a lesser extent.

Walsh [1] developed the first hypothesis about laser analgesia protocol by irradiating the cervical part of the tooth. They proposed the following Er:YAG PPLA protocol: pulse frequency 15–20 Hz; pulse energy 80–120 mJ; water spray setup “on”; focused or slightly defocused beam; contact or non-contact handpiece. This work was followed by a publication in 2008 [3] highlighting that energy should be directed at the CEJ towards the dental pulp through the enamel or dentine by moving the laser hand-piece in a sweeping motion approximately 2–3 mm/s for 20–30 s (exposure time). However, the tip-to-tissue distance was not reported. The authors claimed that the optimal effect was gained at a pulse frequency of 20 Hz, which appeared to align with the resonance frequency needed to temporarily disrupt Na/K pump action. The scientific literature led us to conclude that to the best of our knowledge, there is a lack of scientific evidence of the real bio-resonance frequency of the nerve fibres in the pulp.

Interestingly, a clinical trial conducted by Genovese et al. [10] utilised Er:YAG to evaluate the pain self-reported by paediatric patients on a visual analogue scale (VAS) during cavity preparation, in which the following laser protocol was utilised: power output: 0.5 W and then increased to 2 W, pulse frequency: 20 Hz, pulse energy: 75 mJ, laser tip-to-tissue distance: 3 mm, defocused mode on the gingival margin (1–3 mm) and then slowly moved for 2 min. The usage of water cooling during the procedure was not reported. Additionally, there was a lack of substantial information on the duration and the steps in which the energy was applied, how it was increased and whether any water was used as a cooling agent during the manipulation. Due to the lack of reported laser parameters and protocol, it is difficult to reproduce this protocol for future studies.

Many studies investigated the clinical efficacy of various modifications of the laser analgesia technique with the help of an electrical pulp tester (EPT), using a 904 nm GaAs diode laser [9] or 1064 nm Nd: YAG [6] without previous verification of the proposed protocols’ safety.

To the best of our knowledge, a study conducted by Chan et al. [7] showed the first SEM investigation on teeth irradiated with the following modified laser analgesia protocol of Nd:YAG: average power: 1.1 ± 0.2 W, power density: 3.9 ± 0.7 W/cm^2^, spot area: 0.28 cm^2^, pulse frequency: 15 Hz, fluence: 0.260 ± 0.047 J/cm^2^. They concluded that no alteration to the mineralised tooth structure was induced [8]. However, in a previous clinical study conducted by Chan et al., using the same laser settings reported, the tip was placed approximately 1 mm from the tooth surface. Nevertheless, the tip-to-tissue distance was not reported in this investigation.

A clinical study conducted by Poli et al. [19] developed the following modified laser analgesic protocol using Er,Cr:YSGG (2780 nm): power output increased from 0.1 W to 1 W (15% water, 20% air), fluence: between 6 and 59 J/cm^2^, tip-to-tissue distance: 10 mm, buccal and lingual cervical areas irradiated for 3 min and 30 s. They evaluated self-reported pain during cavity preparation and EPT threshold. However, their study did not provide any information about the morphological alterations on the enamel surface, despite the fact that the laser energy was applied on the neck of the tooth and on the occlusal surface using a Rabbit technique, which is based on utilising high-energy laser-induced analgesia [4]. However, our results have demonstrated that the structural changes were consistent with the applied fluence and water level. Our laser protocol of 0.6 W at a fluence of 10 J/cm^2^ with maximum water cooling demonstrated a sub-ablative laser action achieved at a distance of 10 mm. However, higher power settings and lower water-cooling levels corresponded to an increase in the enamel roughness and impact surface cratering. Hence, laser parameters equal to or greater than the average power of 0.75 W, fluence 12 J/cm^2^ (without or with maximum water cooling) provide an ablation, leading to morphological changes in the enamel surface, which is in disagreement with Poli et al.’s protocol [19].

Our results have shown through SEM that the structural changes in the enamel surfaces were consistent with the applied energy densities and with 100% of water or without water spray. Higher power settings with no water cooling corresponded to an increase in the enamel roughness and impact surface cratering. Laser parameters equal to or greater than an average power of 0.75 W and fluence of 12 J/cm^2^ (without or with maximum water cooling) provided an ablation, which led to morphological changes in the enamel surface. On the other hand, there was no alteration to the mineralised tooth structure with the experimental parameters with 100% water spray or without water spray when the following protocol was applied on the irradiated area fixed at a 10 mm tip-to-tissue distance: 0.2 W/10 Hz/3 J/cm^2^. Our data have shown that a fluence between 10 and 12 J/cm^2^ was considered optimal without any enamel morphological changes.

Our study aimed to find the highest possible sub-ablative energy and water settings, given the limitations of our laser device, without damaging the enamel surface. Our results have shown that the following PPLA protocol was non-ablative to the cervical enamel surface with or without water: 0.2 W/10 Hz/3 J/cm^2^, laser tip-to-tissue distance of 10 mm. Additionally, a sub-ablative laser action at a distance of 10 mm can be achieved when the following protocol is utilised: 0.6 W/15 Hz/10 J/cm^2^ with maximum water cooling. However, the higher energy levels of the protocols 0.75 W/15 Hz/12 J/cm^2^ and 1 W/20 Hz/17 J/cm^2^ have proven to be ablative even when the maximum water spray was used, showing a scaly surface and impact craters with exposed enamel prisms in the SEM investigation.

In light of the above-mentioned statements and our present study, our null hypothesis was not rejected.

## 5. Conclusions

The data of our ex vivo study confirm for the first time that the following Er:YAG sub-ablative PPLA protocol causes no morphological changes in the enamel surface: average power output: 0.6 W, fluence: 10 J/cm^2^, maximum water cooling of 100%, tip-to-tooth tissue: 10 mm, 30 s exposure time, 1.3 × 6.3 mm sapphire tip, sweeping motion with 2 mm/s speed of movement. Additional clinical studies utilising our protocols are warranted to validate Er:YAG laser analgesic effects for painless laser-assisted restorative treatments.

## Figures and Tables

**Table 1:** The distribution of the pre-emptive pulp laser analgesia (PPLA) parameters according to the experimental group of the study treatment protocol.

Laser Parameters	Group A	Group B	Group C	Group D	Group E	Group F	Group G	Group I
Average power (W)	0.2	0.2	0.6	0.6	0.75	0.75	1	1
Pulse frequency (Hz)	10	10	15	15	15	15	20	20
Energy per pulse (mJ)	20	20	40	40	50	50	50	50
Fluence (J/cm^2^)	3	3	10	10	12	12	17	17
Water spray (%)	100	0	100	0	100	0	100	0
Tip-to-tissue distance	10 mm							
Speed of movement	2 mm/s							
Tip type and diameter	1.3 × 6.3 mm sapphire tip							
Irradiation time	30 s/sample							

## Data Availability

All the data are available in the text.
